# Real-World Survival Outcomes of First-Line Therapies in Patients with Metastatic Clear Cell Renal Cell Carcinoma: A Retrospective Analysis from Two Centres in Saudi Arabia

**DOI:** 10.3390/cancers16183234

**Published:** 2024-09-23

**Authors:** Mubarak M. Al-Mansour, Syed Sameer Aga, Hanin A. Alharbi, Maria N. Alsulami, Halah A. Fallatah, Tarfah B. Albedaiwi, Lujain K. Anbari, Taleen R. Surrati, Ashwag A. Algethami, Alaa Althubaiti, Turki M. Alfayea, Ashwaq Alolayan

**Affiliations:** 1 Adult Medical Oncology, Princess Noorah Oncology Centre, Ministry of National Guard Health Affairs-Western Region (MNGHA-WR), King Abdullah International Medical Research Centre (KAIMRC), King Saud Bin Abdulaziz University for Health Sciences (KSAU-HS), King Abdulaziz Medical City, Jeddah 21423, Saudi Arabia; 2 Basic Medical Sciences, College of Medicine, King Saud Bin Abdulaziz University for Health Sciences (KSAU-HS), King Abdullah International Medical Research Centre (KAIMRC), King Abdulaziz Medical City, Jeddah 21423, Saudi Arabia; 3 Department of Clinical Pharmacy, Pharmaceutical Care Services, Ministry of National Guard Health Affairs-Western Region (MNGHA-WR), King Abdullah International Medical Research Centre (KAIMRC), King Abdulaziz Medical City, Jeddah 21423, Saudi Arabia; 4 Adult Medical Oncology Department, Ministry of National Guard Health Affairs-Central Region (MNGHA-CR), King Abdullah International Medical Research Centre (KAIMRC), King Saud Bin Abdulaziz University for Health Sciences (KSAU-HS), King Abdulaziz Medical City, Riyadh 11481, Saudi Arabia

**Keywords:** renal cell cancer, metastasis, immunotherapy, survival

## Abstract

**Simple Summary:**

In this retrospective study, we compared the survival outcomes of first-line ICI regimens versus single-agent TKIs in patients with mRCC from two centres in Saudi Arabia. The patients receiving ICI regimens exhibited improved progression-free survival (PFS) and overall survival (OS) compared to those treated with TKIs. The choice of first-line therapy significantly improves the survival outcomes, underscoring the importance of personalized treatment approaches in managing mRCC. The findings emphasize the significance of baseline patient characteristics in tailoring treatment strategies for mRCC. This research contributes valuable insights into optimizing treatment strategies for mRCC, aiming to enhance patient outcomes in clinical practice.

**Abstract:**

**Background**: Metastatic renal cell carcinoma (mRCC) represents a challenging condition characterised by poor prognosis and limited response to chemoradiotherapy. In this retrospective study, we compared the survival outcomes of first-line ICI regimens versus single-agent TKIs in patients with mRCC from two centres in Saudi Arabia. **Methods**: This study included 84 patients diagnosed with clear cell mRCC between January 2016 and December 2023. Patients were grouped based on treatment regimens. Progression-free survival (PFS) and overall survival (OS) were analysed using Kaplan–Meier curves and Cox proportional hazards regression. **Results**: The median first-line PFS was 9.7 months (95% CI: 5.3–14.1) for the overall cohort, with no significant difference between the single-agent tyrosine kinase inhibitor (TKI) group (9.4 months; 95% CI: 6.4–12.4), combination ICI group (9.0 months; 95% CI: 0.0–24.9), and single-agent ICI group (21.2 months; 95% CI: 2.6–39.8; *p* = 0.591). The median OS for the overall cohort was 42.0 months (95% CI: 14.9–69.2), with the single-agent TKI group having a median OS of 33.3 months (95% CI: 0.0–71.7), the combination ICI group, 42.0 months (95% CI: 0.06–84.0), and the single-agent ICI group, 23.0 months (95% CI: 19.2–26.7; *p* = 0.73). In comparison, the ICI-based combination therapy group exhibited a higher ORR of 41.0% (95% CI: 26.3–57.8%), while the single-agent ICI group had an ORR of 20.0% (95% CI: 3.5–55.8%). Cox regression identified liver metastasis as a significant independent predictor of PFS (HR = 1.8, *p* = 0.043), while a lower Karnofsky Performance Status was a significant independent predictor of OS (HR = 3.5, *p* < 0.001). **Conclusions**: In real-world practice from Saudi Arabia, first-line, single-agent ICI therapy offers promising anti-tumour activity and non-inferior survival outcomes compared to standard ICI-based combinations and single-agent TKIs.

## 1. Introduction

Renal cell carcinoma (RCC) accounts for approximately 2–3% of all cancers, with clear cell carcinoma being the most common subtype [1]. Previous statistics estimated nearly 431,000 new cases of RCC in 2022 globally, with an estimated 180,000 related deaths worldwide [2]. Metastatic renal cell carcinoma (mRCC) represents a challenging condition characterised by poor prognosis and limited response to chemoradiotherapy. Nearly 35% of the patients with RCC show unresectable or metastatic (mRCC) disease at diagnosis [3]. The survival outcomes of mRCC have shown little variation until the introduction of targeted therapy, which has resulted in a positive shift in the overall survival (OS) of patients with mRCC [4,5]. In 2005, the U.S. Food and Drug Administration (FDA) approved the first tyrosine kinase inhibitor (TKI), sorafenib, for mRCC [6]. Since then, several targeted therapies have been approved, expanding the treatment landscape of mRCC [7]. However, mRCC patients eventually develop resistance to target agents, leading to a high rate of treatment failure and poor long-term OS (5-year OS = 12%) [8,9].

The evolution of immune checkpoint inhibitors (ICIs) has resulted in a paradigm shift in mRCC management and improved patient outcomes despite its initial modest effects in the 1990s [9,10,11]. The initial breakthrough in the use of ICIs for mRCC came with the approval of nivolumab, a PD-1 inhibitor, which demonstrated significant improvements in progression-free survival (PFS) and OS compared to everolimus, a mammalian target of rapamycin (mTOR) inhibitors in patients with previously treated mRCC [12]. Subsequently, pivotal trials have consistently shown that ICI-based combinations—such as nivolumab and ipilimumab, pembrolizumab with axitinib, and avelumab with axitinib—significantly improve the treatment response and OS compared to TKIs or mTOR inhibitors in the first-line settings [13,14]. Given these data, the current clinical guidelines recommend ICI-based combinations for first-line management of mRCC with intermediate or poor risk [15]. Recent phase-II trials also demonstrated that single-agent ICIs had promising anti-tumour activities in treatment-naïve patients [16].

Despite the well-established survival benefits of ICI-based regimens in clinical trials and guideline recommendations, there are significant differences between trial results and real-world treatments in the context of mRCC. Clinical trials often have stringent inclusion criteria, controlled settings, and intensive patient monitoring, which may not fully represent the broader, more diverse patient populations encountered in everyday clinical settings. Recent studies have also suggested that ICI clinical trials have historically underrepresented minority populations [17]. Furthermore, the influence of race and ethnicity on mRCC outcomes has become an increasingly recognised factor [18,19]. Thus, real-world studies are crucial to understanding the survival outcomes of ICI-based regimens in mRCC patients with diverse characteristics.

In Saudi Arabia, renal cancers account for nearly 3% of all new cancer cases (85–90% are RCC) [20]. Recent data suggest that the incidence of RCC has notably increased in Saudi Arabia over the past few decades, significantly increasing the burden of mRCC in the kingdom [21]. Despite the rising burden, there is limited evidence about the outcomes of patients with mRCC in Saudi Arabia, especially in the era of ICI. In this retrospective investigation, we compared the survival outcomes of first-line ICI regimens to single-agent TKIs in patients with mRCC from two Saudi Arabian centres.

## 2. Materials and Methods

This study was conducted in accordance with the principles outlined in the Declaration of Helsinki. The retrospective chart review was approved by the Institutional Review Boards (IRBs) of both participating centres. Given the retrospective nature of the study, a waiver of informed consent was obtained from the IRBs.

### 2.1. Data Source and Population

This retrospective chart review included patients diagnosed with clear cell mRCC between January 2016 and December 2023 at Princess Norah Oncology Centre in Jeddah and the Oncology Department at National Guard Hospital in Riyadh. Eligible patients were identified through electronic medical records (EMRs) maintained at both institutions. Inclusion criteria were as follows: adult patients (≥18 years) with histologically confirmed clear cell mRCC who received first-line systemic therapy for mRCC during the data collection period (2016–2023). Patients were excluded if they had non-clear cell histology, had received prior systemic therapy for mRCC, or had incomplete medical records or insufficient follow-up data. Eligible patients were categorised into one of the following treatment groups: Group 1 (G1), patients treated with single-agent TKIs, such as sunitinib, cabozantinib, or pazopanib; Group 2 (G2), patients treated with a combination of ICI and TKIs, including pembrolizumab plus lenvatinib, nivolumab plus cabozantinib, or pembrolizumab plus axitinib; and Group 3 (G3), patients treated with single-agent ICI, such as pembrolizumab or nivolumab.

Patients were included using a non-probability consecutive sampling technique to collect the records of all eligible patients during the data collection period. Data extracted from the EMRs included demographic information (age, sex, and body mass index [BMI]), clinical characteristics (stage, International Metastatic RCC Database Consortium [IMDC], Karnofsky Performance Status [KPS], Memorial Sloan Kettering Cancer Centre [MSKCC], and metastasis sites), treatment details (treatment lines, prior surgery, and adjuvant therapies), treatment response according to the Response Evaluation Criteria in Solid Tumours (RECIST) 1.1 criteria, PFS, and OS. Patients were followed up till death or loss of follow-up.

### 2.2. Statistical Analysis

Statistical analysis was performed using SPSS version 29.0 (IBM Corp., Armonk, NY, USA). Descriptive statistics were used to summarise the characteristics of the study population, including median and interquartile range (IQR) for continuous variables and frequencies and percentages for categorical variables. Comparisons between the three treatment groups were conducted using the Chi-square test for categorical variables and Kruskal–Wallis test for continuous variables. The survival analysis was carried out to assess PFS and OS. PFS was defined as the time from the initiation of treatment to the date of disease progression or death from any cause, whichever occurred first. OS was defined as the time from the initiation of treatment to the date of death from any cause. Kaplan–Meier survival curves were generated for both PFS and OS, and the log-rank test was used to compare survival distributions between the treatment groups. Cox proportional hazards regression analysis was conducted to identify independent predictors of PFS and OS. Variables included in the multivariate analysis were those with a *p*-value < 0.1 in univariate analysis and clinically relevant variables, such as age, sex, performance status, comorbidities, and treatment regimen. We evaluated the proportional hazards (PH) assumption for the Cox regression models using Schoenfeld residuals to ensure the validity of the model. The results indicated that the PH assumption was not violated for the covariates included in our analysis. Additionally, we explored the potential use of time-dependent covariates but did not find any significant time-varying effects within our dataset. Hazard ratios (HRs) and 95% confidence intervals (CIs) were calculated for each variable. All statistical tests were two-sided, and results were considered significant at a *p*-value of less than 0.05.

## 3. Results

Eighty-four patients were included in the present study. Of them, 35 patients received single-agent TKIs, 39 received ICI-based combinations, and 10 received single-agent ICI. The median (IQR) age of patients was significantly different among the groups, with those receiving single-agent ICI being the oldest (67.5 [60.8–79.3] years) compared to those on single-agent TKIs and combination therapy (*p* = 0.019). On the other hand, there were no significant differences between the treatment groups in terms of gender distribution (*p* = 0.583), BMI (*p* = 0.166), KPS (*p* = 0.552), MSKCC criteria (*p* = 0.960), IMDC criteria (*p* = 0.875), initial staging of RCC (*p* = 0.332), or tumour location (*p* = 0.868). Likewise for the distribution of metastatic sites (*p* = 0.420), prior nephrectomy (*p* = 0.100), and surgical type (*p* = 0.090) across the three treatment groups. The median follow-up for the study cohort was 16.5 (7–36.8) months. There were no significant differences between the treatment groups in terms of follow-up duration (*p* = 0.529, Table 1).

### 3.1. Treatment Characteristics and Responses

The distribution of lines of therapy showed that 40.0% of patients on single-agent TKIs, 64.1% of those on combination therapy, and 80.0% of those on single-agent immunotherapy received only one line of therapy (*p* = 0.099). On the other hand, 8.6% and 10.3% of the patients in the single-agent TKI and ICI-based combination therapy groups, respectively, received four lines of therapy. As the first-line treatment, the most commonly administered TKI in the single-agent TKI group was sunitinib (68.6%), followed by pazopanib (28.6%). The ICI-based combination therapy group predominantly received ipilimumab and nivolumab (38.5%), pembrolizumab and lenvatinib (30.8%), and pembrolizumab and axitinib (20.5%). In the single-agent ICI group, nivolumab (50.0%) and pembrolizumab (50.0%) were the drugs administered. The response rates to first-line therapy varied significantly among the groups (*p* = 0.013). In the single-agent TKI group, 71.4% of patients had progressive disease (PD) compared to 51.3% in the ICI-based combination group and 50% in the single-agent ICI group (Table 2). The objective response rate (ORR) for the single-agent TKI group was 17.1% (95% CI: 7.2–32.1%). In comparison, the ICI-based combination therapy group exhibited a higher ORR of 41.0% (95% CI: 26.3–57.8%), while the single-agent ICI group had an ORR of 20.0% (95% CI: 3.5–55.8%).

In the second line, most patients in the single-agent TKI group received nivolumab (76.2%). In comparison, the most commonly administered second-line drug was cabozantinib (42.9%), followed by sunitinib (28.6%) in the ICI-based combination group. The single-agent ICI group received pazopanib (100%). Response rates to second-line therapy did not differ significantly across the treatment group (*p* = 0.485). The same findings were observed at the third (*p* = 0.915) and fourth lines (*p* = 0.797), Table 2.

### 3.2. Survival Outcomes

**Figure 1:**
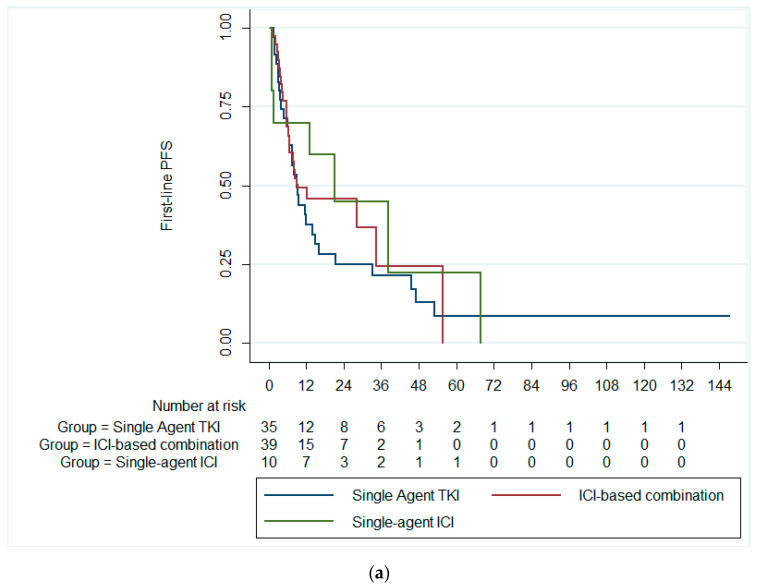

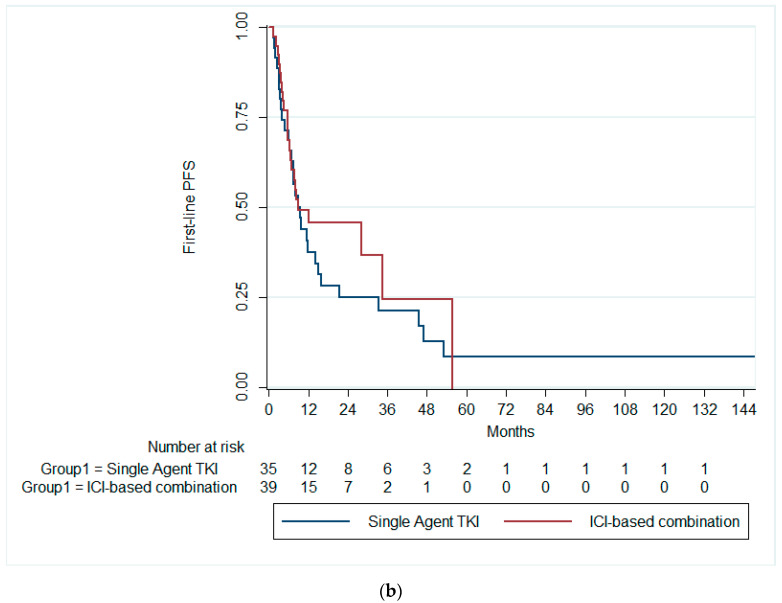
Kaplan–Meier curves of the PFS (**a**) across the three treatment groups and (**b**) in patients receiving ICI-based combinations and single-agent TKI. PFS, progression-free survival; ICI, immune checkpoint inhibitors; TKI, tyrosine kinase inhibitor.

The median first-line PFS was 9.7 months (95% CI: 5.3–14.13). There was no statistically significant difference in the median first-line PFS among patients receiving single-agent TKI (median PFS = 9.4 months; 95% CI: 6.4–12.4), combination ICIs (median PFS = 9.0 months; 95% CI: 0.0–24.9), and single-agent ICI (median PFS = 21.2 months; 95% CI: 2.6–39.8; *p* = 0.591), Figure 1a. Likewise, there was no significant difference in the median first-line PFS between patients receiving single-agent TKI and combination ICIs (*p* = 0.369), Figure 1b.

**Figure 2:**
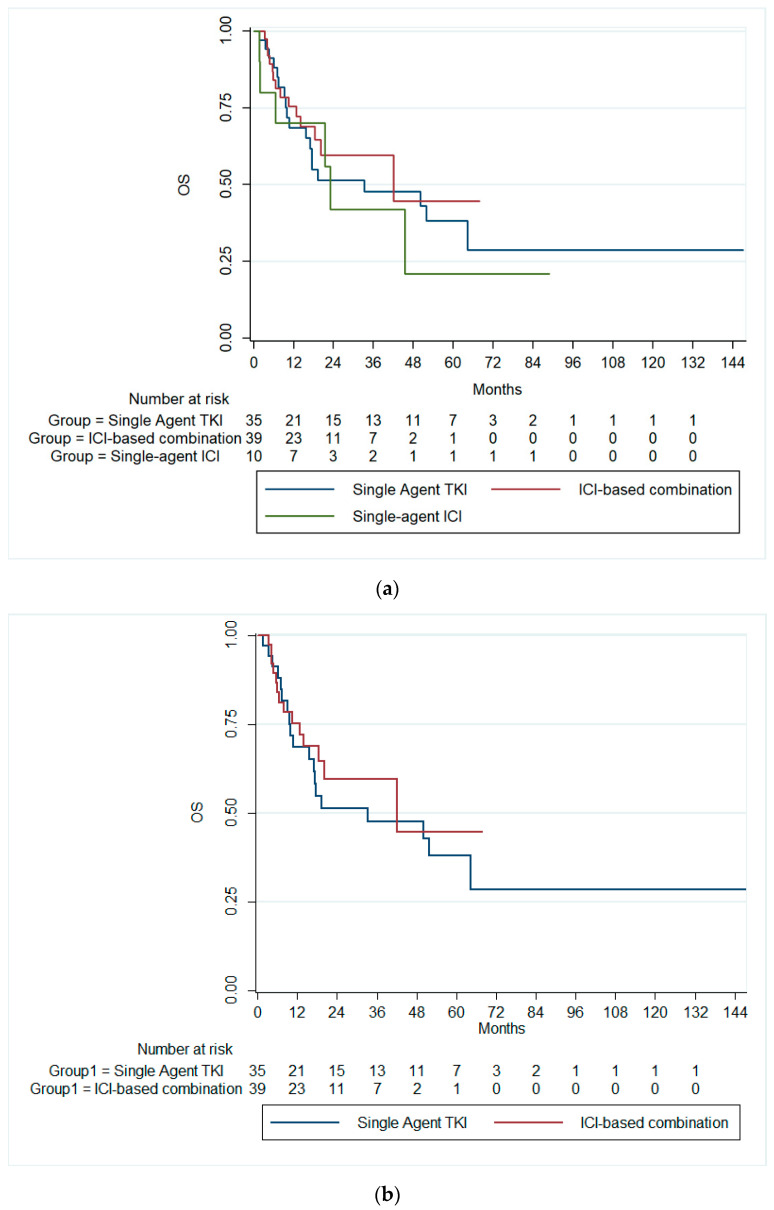
Kaplan–Meier curves of the OS (**a**) across the three treatment groups and (**b**) in patients receiving ICI-based combinations and single-agent TKI. OS, overall survival; ICI, immune checkpoint inhibitors; TKI, tyrosine kinase inhibitor.

On the other hand, the median OS for the overall cohort was 42 months (95% CI: 14.9–69.2). Patients receiving single-agent TKIs exhibited a median OS of 33.3 months (95% CI: 0.0–71.7). Those treated with combination immunotherapy had a median OS of 42.0 months (95% CI: 0.06–84.0), while patients on single-agent immunotherapy demonstrated a median OS of 23.0 months (95% CI: 19.2–26.7). There was no significant difference in the median OS across all therapy groups (*p* = 0.728), Figure 2a. Likewise, there was no significant difference in the median OS between patients receiving single-agent TKI and combination ICIs (*p* = 0.589), Figure 2b.

### 3.3. Predictors of Survival Outcomes

The univariate Cox regression analysis showed that a KPS score < 80 (HR = 2.4, 95% CI: 1.4–4.3, *p* = 0.002), high-risk MSKCC (HR = 11.1, 95% CI: 2.4–50.9, *p* = 0.002), high-risk IMDC (HR = 10.7, 95% CI: 2.4–48.4, *p* = 0.002), and liver metastasis (HR = 2.0, 95% CI: 1.2–3.5, *p* = 0.015) were significantly associated with first-line PFS. In the adjusted model, only liver metastasis remained a significant predictor of first-line PFS (HR = 1.8, 95% CI: 1.1–3.3, *p* = 0.043), Table 3.

For the OS, the univariate Cox regression analysis showed that a KPS score < 80 (HR = 4.2, 95% CI: 2.1–8.1, *p* < 0.001), prior nephrectomy (HR = 0.49, 95% CI: 0.25–0.94, *p* = 0.032), and bone metastasis (HR = 2.01, 95% CI: 1.07–3.79, *p* = 0.031) were significantly associated with OS. In the adjusted model, a KPS score < 80 remained significant with an HR of 3.5 (95% CI: 0.16–7.4, *p* < 0.001). Liver metastasis approached significance with an HR of 1.89 (95% CI: 0.93–3.87, *p* = 0.078) [Table 4].

## 4. Discussion

In this study, we evaluated the survival outcomes and treatment responses of the first-line therapies in patients with mRCC. Our findings reveal that first-line single-agent ICIs were not inferior to ICI-based combinations and single-agent TKIs regarding PFS and OS. Additionally, the treatment response at the first line was significantly better in the ICI single-agent and ICI-based combination groups, in which fewer patients had PD than single-agent TKIs. On the other hand, we identified significant predictors of PFS and OS in real-world practice. The single-agent ICI was also associated with comparable ORR to single-agent TKI, while the ICI-based combinations were associated with notably higher ORR. A lower KPS score and the presence of liver metastasis were consistently associated with poorer outcomes.

Historically, mRCC was associated with dismal survival outcomes prior to the advent of targeted therapies, with a median OS of approximately 13 months and a 2-year OS rate of less than 20% [22]. However, the therapeutic landscape of mRCC began to shift dramatically with the introduction of TKIs, which extended the median OS to approximately 26 months [23]. Despite these advancements, many mRCC patients eventually developed resistance to TKIs, leading to treatment failure and a poor long-term OS (5-year OS = 12%) [8,9]. The evolution of ICIs represented a new frontier in mRCC treatment. ICI’s pivotal trials have consistently shown that ICI-based combinations, such as nivolumab and ipilimumab, pembrolizumab with axitinib, and avelumab with axitinib, significantly improved treatment responses and OS compared to TKIs or mTOR inhibitors in the first-line setting [13,14]. In the present study, we noted that the overall PFS and OS of the mRCC patients from Saudi Arabia during the era of targeted therapies were 9.7 months (95% CI: 5.3–14.13) and 42 months (95% CI: 14.9–69.2), respectively. Such estimates appear to reflect a notable improvement in the survival outcomes of patients with mRCC compared to the pre-targeted therapies era. In a retrospective analysis from Saudi Arabia that collected the mRCC patients’ data between 2003 and 2013, the median OS was 2.03 years (95% CI: 1.8–2.3) [21].

Despite their well-established efficacy, ICI-based combinations can significantly increase the burden of toxicities compared to single agents, particularly when combined with vascular endothelial growth factor (VEGF)-TKI. In a recent systematic review and meta-analysis, patients with mRCC receiving ICI-based combination had fewer haematological toxicities compared to TKIs; however, they exhibited higher rates of hepatotoxicity, nephrotoxicity, and gastrointestinal symptoms. The higher toxicities were more evident in patients receiving ICI plus VEGF-TKI [24]. Other reports showed similar findings [25,26]. Thus, recent studies have evaluated the outcomes of single-agent ICIs in first-line settings to improve the tolerability profile of first-line therapy, particularly in patients with high comorbidities and those who cannot tolerate ICI-based combinations [16]. The present study found that the first-line single-agent ICIs were not inferior to ICI-based combinations and single-agent TKIs with regard to PFS and OS. In the KEYNOTE-427 phase-II single-arm trial, which evaluated pembrolizumab monotherapy in treatment-naïve patients with advanced clear cell RCC, the median PFS was 7.1 months, with a 2-year OS of 71% [16]. In non-clear cell patients, the median PFS was lower (median 4.2 months), with a 2-year OS rate of 58% [27].

The present study also showed that single-agent ICI was not inferior to single-agent TKIs in terms of treatment response. The ORR for the single-agent TKI group was 17.1% (95% CI: 7.2–32.1%) compared to 20.0% (95% CI: 3.5–55.8%) in the single-agent ICI. Additionally, in the single-agent TKI group, 71.4% of patients had PD compared to 51.3% in the ICI-based combination group and 50% in the single-agent ICI group. While data from our study confirms the more robust anti-tumour activity of ICI-based combinations in line with pivotal trials [13,14], it also shows the comparable anti-tumour activity of single-agent ICI to single-agent TKIs, highlighting their potential as a viable first-line treatment option, particularly for patients who may not tolerate combination therapies. In the KEYNOTE-427 trial, the ORR was 36.4% in clear cell mRCC [16] and 26.7% in non-clear cell ORR [27]. The lower ORR observed in our study may be attributed to the small sample size of the single-agent ICI cohort.

TKIs such as sunitinib and pazopanib are known to be associated with a range of adverse effects, including hypertension, hand–foot syndrome, fatigue, diarrhoea, and haematologic toxicities, which can be severe and may necessitate dose reductions or discontinuation of therapy [28]. On the other hand, while ICI-based combinations do not increase the risk of severe or fatal adverse events compared to single-agent TKIs [24,29], the combination of ICIs with TKIs in combination regimens introduces additional complexities in managing overlapping toxicities, especially in patients with high comorbidity burden, increasing treatment burden and cost. Previous real-world studies showed that combining ICI and TKI led to grade ≥3 adverse events in 43.1% of the patients and a discontinuation rate of 27% [30,31]. While the present study did not evaluate the safety profile of different treatment regimens, the KEYNOTE-427 trial observed that pembrolizumab monotherapy was associated with lower rates of treatment-related adverse events and treatment discontinuation than the reported rates of ICI-based combinations in pivotal trials [16,27].

The present study is one of the few published reports about the survival outcomes of first-line therapies for mRCC in a real-world setting in Saudi Arabia; however, we acknowledge the existence of certain limitations. Firstly, the sample size, particularly for the single-agent ICI group, was relatively small (*n* = 10), which may limit the generalisability of the findings and the statistical power to detect significant differences among treatment groups. Secondly, this was a retrospective analysis, which is inherently subject to selection bias. The retrospective nature of the study also limited our ability to control for all potential confounding variables. Despite our efforts to adjust for confounding factors using multiple statistical adjustments, these methods cannot fully eliminate the risk of selection bias due to the heterogeneity of the patient population. The heterogeneity in patient characteristics and treatment regimens, such as variations in the type of TKIs and ICIs used, could have influenced the outcomes. While we adjusted for several known prognostic factors, other unmeasured variables could have influenced survival outcomes, such as genetic mutations, molecular markers, and prior lines of therapy. Due to the retrospective nature of our study, causal relationships between the variables studied and patient outcomes cannot be definitively established. Additionally, the reliance on medical records for data collection could introduce inaccuracies due to incomplete or inconsistent documentation. The different follow-up durations and treatment modifications over time might also have impacted the results. Lastly, the safety profiles of the treatments were not evaluated in this study. Future studies should include comprehensive safety evaluations to provide a more holistic understanding of the benefits and risks associated with these therapies. There is a need for further multi-centre studies with larger cohorts to validate our results and enhance generalisability.

## 5. Conclusions

In real-world practice from Saudi Arabia, first-line single-agent ICI offers promising anti-tumour activities and non-inferior survival outcomes compared to the standard ICI-based combinations and single-agent TKIs. Our results suggest that single-agent ICI offers a durable tumour response. However, due to the limited sample size, we could not assess the survival outcomes across different subgroups of IMD and KPS, which warrants further investigation. Our study also identified critical predictors of PFS and OS among Saudi patients with mRCC in real-world practice, including lower KPS and liver metastasis. These findings highlight the importance of baseline patient characteristics in guiding treatment decisions and personalised treatment strategies in RCC. Larger multi-centre trials are needed to confirm our findings and identify biomarkers that predict response in patients with mRCC.

## Figures and Tables

**Table 1:** Demographics and clinical characteristics.

Characteristics		Total (*n* = 84)	Single-Agent TKI (*n* = 35)	Combination Therapy (*n* = 39)	Single-Agent ICI (*n* = 10)	*p*-Value
**Age**	Median (IQR)	60.5 (54–68.8)	61 (56–69)	58 (52–65)	67.5 (60.8–79.3)	0.019
**Gender**	Female	26 (31.0%)	12 (34.3%)	10 (25.6%)	4 (40.0%)	0.583
	Male	58 (69.0%)	23 (65.7%)	29 (74.4%)	6 (60.0%)	
**BMI**	Median (IQR)		26.3 (22.7–28.8)	23.5 (20.4–31.1)	25.7 (22.8–36.4)	0.166
**Karnofsky performance status score**	>80	61 (72.6%)	25 (71.4%)	30 (76.9%)	6 (60.0%)	0.552
	<80	23 (27.4%)	10 (28.6%)	9 (23.1%)	4 (40.0%)	
**MSKCC Prognostic risk group**	Favourable	4 (4.8%)	2 (5.7%)	2 (5.1%)	0 (0.0%)	0.960
	Intermediate	50 (59.5%)	21 (60.0%)	23 (59.0%)	6 (60.0%)	
	High	30 (35.7%)	12 (34.3%)	14 (35.9%)	4 (40.0%)	
**IMDC prognostic risk group**	Favourable	5 (6.0%)	2 (5.7%)	3 (7.7%)	0 (0.0%)	0.875
	Intermediate	46 (54.8%)	20 (57.1%)	21 (53.8%)	5 (50.0%)	
	Poor	33 (39.3%)	13 (37.1%)	15 (38.5%)	5 (50.0%)	
**Initial staging of RCC**	Stage 1	1 (1.2%)	1 (2.9%)	0 (0.0%)	0 (0.0%)	0.332
	Stage 2	6 (7.1%)	5 (14.3%)	1 (2.6%)	0 (0.0%)	
	Stage 3	14 (16.7%)	6 (17.1%)	7 (17.9%)	1 (10.0%)	
	Stage 4	63 (75.0%)	23 (65.7%)	31 (79.5%)	9 (90.0%)	
**Location**	Unilateral	82 (97.6%)	34 (97.1%)	38 (97.4%)	10 (100.0%)	0.868
	Bilateral	2 (2.4%)	1 (2.9%)	1 (2.6%)	0 (0.0%)	
**Number of sites of metastasis**	1	31 (36.9%)	13 (37.1%)	13 (33.3%)	5 (50.0%)	0.420
	2 or more	53 (63.1%)	22 (62.9%)	26 (66.7%)	5 (50.0%)	
**Nephrectomy**	No Prior Nephrectomy	40 (47.6%)	11	22	7	0.100
	Previous Nephrectomy	43 (51.2%)	23	17	3	
	N/R	1 (1.2%)	1	0	0	
**Type of surgery**	Partial Nephrectomy	2 (2.4%)	0	2	0	0.090
	Radical Nephrectomy	41 (48.8%)	23	15	3	
	N/A	40 (47.6%)	11	22	7	
	N/R	1 (1.2%)	1	0	0	
**Adjuvant therapy (Sunitinib)**	No Prior Adjuvant Therapy	83 (98.8%)	35 (100.0%)	39 (100.0%)	9 (90.0%)	0.024
	Prior Adjuvant Therapy	1 (1.2%)	0 (0.0%)	0 (0.0%)	1 (10.0%)	
**Metastatic site**	Lung	59 (70.2%)	22 (62.9%)	30 (76.9%)	7 (70.0%)	0.418
	Liver	23 (27.4%)	10 (28.6%)	11 (28.2%)	2 (20.0%)	0.855
	Lymph Node	32 (38.1%)	11 (31.4%)	17 (43.6%)	4 (40.0%)	0.556
	Brain	9 (10.7%)	2 (5.7%)	6 (15.4%)	1 (10.0%)	0.405
	Bone	34 (40.5%)	15 (42.9%)	14 (35.9%)	5 (50.0%)	0.671
	Other Site	25 (29.8%)	13 (37.1%)	9 (23.1%)	3 (30.0%)	0.418
**Follow-up (months)**	Median (IQR)	16.5 (7–36.8)	17.4 (7.2–51)	16 (7.5–24.6)	19 (8.9–28.8)	0.529

**TKI**: tyrosine kinase inhibitors; **BMI**: Body Mass Index; **MSKCC**: Memorial Sloan Kettering Cancer Centre; **IMDC**: International Metastatic Renal Cell Carcinoma Database Consortium; **ICI**: immune checkpoint inhibitor; **IQR**: interquartile range.

**Table 2:** Treatment characteristics and response.

Variables		Single-Agent TKI (*n* = 35)	Combination Therapy (*n* = 39)	Single-Agent ICI (*n* = 10)
**Lines of therapy**	1	14 (40.0%)	25 (64.1%)	8 (80.0%)
	2	15 (42.9%)	6 (15.4%)	1 (10.0%)
	3	3 (8.6%)	4 (10.3%)	1 (10.0%)
	4	3 (8.6%)	4 (10.3%)	0 (0.0%)
**First-line drug**	Cabozantinib	1 (2.9%)	0 (0.0%)	0 (0.0%)
	Ipilimumab and Nivolumab	0 (0.0%)	15 (38.5%)	0 (0.0%)
	Nivolumab	0 (0.0%)	0 (0.0%)	5 (50.0%)
	Nivolumab and Cabozantinib	0 (0.0%)	4 (10.3%)	0 (0.0%)
	Pazopanib	10 (28.6%)	0 (0.0%)	0 (0.0%)
	Pembrolizumab	0 (0.0%)	0 (0.0%)	5 (50.0%)
	Pembrolizumab and Axitinib	0 (0.0%)	8 (20.5%)	0 (0.0%)
	Pembrolizumab and Lenvatinib	0 (0.0%)	12 (30.8%)	0 (0.0%)
	Sunitinib	24 (68.6%)	0 (0.0%)	0 (0.0%)
**First-line response**	CR	3 (8.6%)	0 (0.0%)	0 (0.0%)
	PR	3 (8.6%)	16 (41.0%)	2 (20.0%)
	SD	4 (11.4%)	2 (5.1%)	3 (30.0%)
	PD	25 (71.4%)	20 (51.3%)	5 (50.0%)
**Second-line drug**	***n***	**=21**	**=14**	**=2**
	Cabozantinib	0 (0.0%)	6 (42.9%)	0 (0.0%)
	Ipilimumab and Nivolumab	0 (0.0%)	1 (7.1%)	0 (0.0%)
	Nivolumab	16 (76.2%)	0 (0.0%)	0 (0.0%)
	Nivolumab and Cabozantinib	2 (9.5%)	1 (7.1%)	0 (0.0%)
	Pazopanib	1 (4.8%)	1 (7.1%)	2 (100%)
	Pembrolizumab	1 (4.8%)	0 (0.0%)	0 (0.0%)
	Pembrolizumab and Lenvatinib	0 (0.0%)	1 (7.1%)	0 (0.0%)
	Sunitinib	1 (4.8%)	4 (28.6%)	0 (0.0%)
**Second-line response**	***n***	**=20**	**=14**	**=2**
	CR	1 (5%)	0 (0.0%)	0 (0.0%)
	PR	5 (25%)	3 (21.4%)	0 (0.0%)
	SD	2 (10%)	1 (7.1%)	0 (0.0%)
	PD	12 (60%)	10 (71.4%)	2 (100%)
**Third-line drug**	***n***	**=6**	**=8**	**=1**
	Cabozantinib	2 (33.3%)	1 (12.5%)	1 (100.0%)
	Ipilimumab and Nivolumab	0 (0.0%)	1 (12.5%)	0 (0.0%)
	Lenvatinib and Everolimus	1 (16.7%)	1 (12.5%)	0 (0.0%)
	Nivolumab	2 (33.3%)	0 (0.0%)	0 (0.0%)
	Nivolumab and Cabozantinib	0 (0.0%)	2 (25%)	0 (0.0%)
	Pazopanib	1 (16.7%)	1 (12.5%)	0 (0.0%)
	Pembrolizumab	0 (0.0%)	1 (12.5%)	0 (0.0%)
	Pembrolizumab and Lenvatinib	0 (0.0%)	1 (12.5%)	0 (0.0%)
**Third-line response**	***n***	**=6**	**=8**	**=1**
	CR	1 (16.7%)	2 (25%)	0 (0.0%)
	PR	0 (0.0%)	1 (12.5%)	0 (0.0%)
	SD	5 (83.3%)	5 (62.5%)	1 (100.0%)
**Fourth-line drug**	***n***	**=3**	**=4**	**=0**
	Cabozantinib	1 (33.3%)	0 (0.0%)	0 (0.0%)
	Lenvatinib and Everolimus	1 (33.3%)	0 (0.0%)	0 (0.0%)
	Everolimus	0 (0.0%)	1 (25%)	0 (0.0%)
	Nivolumab	0 (0.0%)	1 (25%)	0 (0.0%)
	Pembrolizumab and Axitinib	1 (33.3%)	0 (0.0%)	0 (0.0%)
	Pembrolizumab and Lenvatinib	0 (0.0%)	1 (25%)	0 (0.0%)
	Sunitinib	0 (0.0%)	1 (25%)	0 (0.0%)
**Fourth-line response**	***n***	**=3**	**=2**	**=0**
	CR	2 (66.7%)	0 (0.0%)	0 (0.0%)
	PR	1 (33.3%)	1 (50%)	0 (0.0%)
	SD	0 (0.0%)	1 (50%)	0 (0.0%)

**CR**: complete response; **PR**: partial response; **PD**: progressive disease; **SD**: stable disease; **NA**: not available. **TKI**: tyrosine kinase inhibitors.

**Table 3:** Cox proportional hazards regression for first-line PFS.

Variables		Univariate Analysis		Adjusted Model	
	No. of Events	HR (95.0% CI)	*p*-Value	HR (95.0% CI)	*p*-Value
**Age ≥ 65 years old**	22	1.1 (0.62–1.8)	0.824		
**Male gender**	41	1.1 (0.64–1.9)	0.697		
**BMI ≥ 30 Kg/m^2^**	11	0.79 (0.41–1.5)	0.490		
**Karnofsky performance status score < 80**	19	2.4 (1.4–4.3)	0.002	1.3 (0.67–2.5)	0.444
**MSKCC prognostic risk group (Ref: favourable)**					
Intermediate	31	3 (0.69–13.2)	0.140	NE	0.913
High	26	11.1 (2.4–50.9)	0.002	NE	0.925
**IMDC prognostic risk group (Ref: favourable)**					
Intermediate	28	3.1 (0.73–13.7)	0.125	NE	0.900
High	29	10.7 (2.4–48.4)	0.002	NE	0.893
**Bilateral location**	1	1.66 (0.22–12.2)	0.646		
**Prior nephrectomy**	31	0.59 (0.35–1.1)	0.057	0.98 (0.54–1.77)	0.961
**Radical nephrectomy**	30	0.95 (0.13–7)	0.958		
**Adjuvant therapy (Sunitinib)**	1	0.34 (0.043–2.6)	0.224		
**First-line treatment (Ref: Single-agent TKI)**					
ICI combination	23	0.79 (0.46–1.4)	0.428		
Single-agent ICI	7	0.7 (0.3–1.61)	0.400		
**Lung metastasis**	41	1.3 (0.7–2.2)	0.359		
**Liver metastasis**	18	2 (1.2–3.5)	0.015	1.8 (1.1–3.3)	0.043
**Lymph node metastasis**	23	1.1 (0.63–1.8)	0.839		
**Brain metastasis**	5	0.83 (0.33–2.1)	0.676		
**Bone metastasis**	25	1.34 (0.79–2.3)	0.270		

**TKI**: tyrosine kinase inhibitors; BMI: body mass index; **MSKCC**: Memorial Sloan Kettering Cancer Centre; **IMDC**: International Metastatic Renal Cell Carcinoma Database Consortium; **HR**: hazard ratio; **CI**: confidence interval; **ICI**: immune checkpoint inhibitor.

**Table 4:** Cox proportional hazards regression for OS.

Variables		Univariate Analysis		Adjusted Model	
	No. of Events	HR (95.0% CI)	*p*-Value	HR (95.0% CI)	*p*-Value
**Age ≥ 65 years old**	17	1.48 (0.79–2.8)	0.228		
**Male gender**	27	1.1 (0.56–2.2)	0.776		
**BMI ≥ 30 Kg/m^2^**	6	0.72 (0.3–1.7)	0.449		
**Karnofsky performance status score < 80**	17	4.2 (2.1–8.1)	<0.001	3.5 (0.16–7.4)	<0.001
**MSKCC prognostic risk Group (Ref: favourable)**					
Intermediate	19	NE	0.916		
High	20	NE	0.904		
**IMDC prognostic risk Group (Ref: favourable)**					
Intermediate	17	NE	0.913		
High	22	NE	0.900		
**Bilateral location**	1	3.9 (0.5–30.5)	0.192		
**Prior nephrectomy**	18	0.49 (0.25–0.94)	0.032	1.2 (0.27–5.54)	0.794
**Radical nephrectomy**	16	0.26 (0.06–1.17)	0.080	0.55 (0.11–2.74)	0.467
**Adjuvant therapy (Sunitinib)**	1				
**First-line treatment (Ref: Single-agent TKI)**					
ICI combination	14	0.84 (0.41–1.69)	0.624		
Single-agent ICI	6	1.22 (0.49–0.31)	0.668		
**Lung metastasis**	25	1.13 (0.58–2.2)	0.717		
**Liver metastasis**	12	1.8 (0.92–3.61)	0.086	1.89 (0.93–3.87)	0.078
**Lymph node metastasis**	15	1.1 (0.56–2.1)	0.832		
**Brain metastasis**	5	1.4 (0.56–3.79)	0.433		
**Bone metastasis**	22	2.01 (1.07–3.79)	0.031	1.26 (0.62–2.56)	0.516

**TKI**: tyrosine kinase inhibitors; BMI: body mass index; **MSKCC**: Memorial Sloan Kettering Cancer Centre; **IMDC**: International Metastatic Renal Cell Carcinoma Database Consortium; **HR**: hazard ratio; **CI**: confidence interval; **ICI**: immune checkpoint inhibitor.

## Data Availability

The raw data used for this research are available on request from the corresponding author.
